# General practitioners’ awareness, use, and perceptions of social prescribing in Austria

**DOI:** 10.3389/fpubh.2026.1897020

**Published:** 2026-07-15

**Authors:** Christina Radl-Karimi, Michael Paulitsch, Dora E. Haxhija, Nicole Posch, Ulrike Spary-Kainz, Andrea Siebenhofer

**Affiliations:** 1 Institute of General Practice and Evidence-Based Health Services Research, Medical University of Graz, Graz, Austria; 2 Institute of General Practice, Goethe University Frankfurt, Frankfurt am Main, Germany

**Keywords:** community support, general practice, link worker, primary care, public health, social prescriptions

## Abstract

**Introduction:**

Social prescribing (SP) is increasingly recognized as an approach to address patients’ nonmedical needs in primary care, yet evidence on general practitioners’ (GPs’) perspectives in Austria is limited. This study explored GPs’ familiarity with SP, its current use, and perceptions of implementation and impact.

**Methods:**

A cross-sectional online survey was conducted from June to October 2024 among 3,849 Austrian GPs. The questionnaire assessed familiarity with and use of SP, perceptions on implementation, and perceived impact on patient care. Data were analyzed using descriptive and inferential statistics.

**Results:**

A total of 160 GPs responded (4.2%). Although 71.3% were initially unfamiliar with the term SP, more than half reported using some form of it, mainly through informal referrals such as signposting. No associations were found between SP use and GP characteristics. While only 37.5% felt able to adequately address patients’ social needs, 96.3% expressed willingness to adopt SP in their practice. Key barriers included insufficient reimbursement and funding as well as limited referral structures and workforce capacity. Overall, SP was viewed as a valuable approach, particularly for addressing loneliness and mental health issues.

**Conclusion:**

Despite limited familiarity with the formal concept, elements of SP are already embedded in routine practice among Austrian GPs. There is strong interest in adopting more structured SP pathways, particularly with support from link workers, but implementation is hindered by gaps in awareness, referral options, and infrastructure. Strengthening training, service availability, and system-level support will be essential for effective and sustainable integration of SP into primary care.

## 1 Introduction

This paper explores Austrian general practitioners’ (GPs) awareness, use, and perceptions of social prescribing (SP) in primary care.

SP can be described as trusted professionals connecting people with nonmedical social needs—often via a link worker (connector)—to community support services to improve well-being and reduce health inequalities (1). Over time, several referral models have emerged to link patients from primary care to community-based activities, including signposting through leaflets, direct referrals to specific activities, referrals facilitated by link workers, and more holistic approaches in which healthcare professionals provide ongoing individual or group-based support (2, 3).

Approximately one in five consultations in general practice is driven by nonmedical problems such as social, emotional, or practical challenges (4). GPs often lack the time or expertise to recommend appropriate interventions for these issues (5). SP is therefore increasingly recognized as a promising approach to address the growing psychosocial and practical needs of patients, including issues such as social isolation, loneliness, mental health problems, and other social determinants of health that influence patients´ well-being (6, 7). Emerging evidence suggests that SP may improve health and well-being while potentially reducing pressure on primary care professionals and other healthcare services (8).

Originating in the United Kingdom in the late 1990s, SP has been integrated into national policy since 2019 as part of the NHS Long Term Plan for primary care (9, 10). Internationally, several countries are also making progress in adopting SP approaches within their health systems (9, 11). In Austria, SP has been piloted since 2021 through national funding calls to strengthen links between primary care and community-based services by enabling healthcare professionals to refer patients with social needs to local support services and organizations (12). To date, SP has been implemented in 24 primary care settings, including solo/group practices and multiprofessional primary care units, with further scale-up planned (13). Given the policy focus on multiprofessional primary care centers (14), Austrian primary care is a suitable setting for SP, providing continuous, and multiprofessional care.

Austrian evidence on SP primarily comprises practice and implementation guidelines (15), experiences from pilot implementation studies and early-stage evaluations (16) alongside policy and conceptual frameworks developed by national public health institutions (12, 17). Evidence on Austrian GPs’ awareness of SP, their current engagement in SP-related referral practices, and their views on feasibility and impact in routine care remains scarce or not yet explicitly investigated. Such insights are essential for informing future SP implementation of SP and guiding policies to integrate social approaches into routine care. This study therefore examines:

1. GPs’ familiarity with and current use of SP
2. GPs’ perspectives on implementing SP, and
3. GPs’ perceptions of SP’s impact on patient care and social issues encountered in daily practice.

## 2 Methods and materials

### 2.1 Study design and setting

This cross-sectional online survey was designed to explore the awareness, attitudes, and perceptions of GPs regarding SP implementation in Austria.

### 2.2 Recruitment and survey administration

Practicing GPs in Austria were invited to participate using convenience sampling. An invitation to participate was sent to all GPs listed in the National Medical Association with publicly accessible email addresses (*N* = 3,849). A reminder email was sent 3 weeks later to encourage participation. The survey was also distributed via the Austrian Primary Health Care Platform. A total of 263 participants accessed and initiated the survey of whom 191 completed it. After exclusion of 31 non-physicians, 160 participants were included in the final analysis (analytic response rate 4.2%). Response rates are based on the National Medical Association mailing list, which was the primary sampling frame, while additional distribution via the Austrian Primary Health Care platform may have extended reach beyond this denominator. Participation was voluntary. As the study involved no intervention in GPs’ clinical practice, no collection of patient or identifiable personal data, and all data were analyzed anonymously, formal ethical approval was deemed unnecessary. The study was conducted in accordance with data protection regulations [EU GDPR (2016/679), Recital 26 and Article 4 (1)].

### 2.3 Questionnaire

The 29-item questionnaire in German was developed based on a literature review, pilot-tested, and initially administered to German GPs (18). For the Austrian version, the introductory text and items on participant characteristics (practice type and region) were adapted. The full questionnaire is provided in the Supplementary material. Participants were first asked about their familiarity with SP and then given an explanation and example. Items assessed GPs’ familiarity with SP, current practice, perceived meaningfulness, common social issues for which SP could be beneficial, and perceived barriers. The questionnaire included seven yes/no items, one multiple-response item, six 1–4 rating scale items, two single-choice items, and 12 demographic/practice questions.

### 2.4 Statistical analysis

To achieve the study aims, visualizations were produced, and descriptive and inferential statistics were conducted. For easier interpretation of results, response categories for the items using the 1–4 rating scales were dichotomized into “does not apply/rather does not apply” and “rather applies/applies.”

Multilevel logistic regression analysis was performed to examine associations between the stated use of SP (yes/no) as the dependent variable and gender, age, and size of town as independent variables. Age-groups and size of town were dummy-coded due to their categorical/ordinal operationalization. Categories/levels with very small sample sizes (three GPs aged under 35 and one with diverse gender) were excluded, resulting in a sample size of 156. A multilevel approach was used to account for clustering of GPs within federal states, as single-level regression could produce biased estimates if responses systematically vary by federal state. A null model was first specified to calculate a latent scale intraclass correlation (ICC), representing systematic variation between federal states. The multilevel approach was retained and additional independent variables were included if the ICC indicated significant clustering. Statistical significance of independent variables was assessed using *p*-values (alpha = 5%), and their magnitude was evaluated with odds ratios and 95% confidence intervals. Given the exploratory nature of the analysis, no formal *a priori* sample size calculation was conducted.

Analyses were conducted in R (version 4.3.0) via Positron (2025.12.1), using *tidyr* for data transformation, *base R* and *dplyr* for descriptive statistics, *ggplot2* for visualizations, and *lme4* for multilevel regression.

## 3 Results

The survey was conducted between June 25 and October 27, 2024. After excluding incomplete responses, a total of 160 completed questionnaires were included for analysis, resulting in a response rate of 4.2%. The sample was balanced by sex and predominantly comprised experienced GPs. Nearly half worked in academic practices, with most practicing in solo-practice settings. Practices commonly served 750–1,500 patients per quarter and were located in small towns (<5,000 residents) or large cities (>100,000 residents). Full details on participant characteristics are provided in Table 1. Key results are presented below. Full descriptive statistics are reported in the Supplementary Figure 1, Supplementary Tables 1–9.

**Table 1:** Sociodemographic characteristics of participating GPs.

Demographics	*n* = 160	%
Sex		
Female	79	49.4%
Male	80	50.0%
Missing	1	0.6%
Age (years)		
<35	3	1.9%
35–45	27	16.9%
46–55	58	36.3%
>55	72	45.0%
Academic practice		
Yes	79	49.4%
No	81	50.6%
Years in practice		
<5	19	11.9%
5–15	57	35.6%
16–30	43	26.9%
>30	41	25.6%
Practice type		
Single practice	131	81.9%
Group practice	18	11.3%
Primary healthcare unit/network	11	6.9%
Patients per quartal		
<750	34	21.3%
750–1,500	63	39.4%
1,500–2,500	48	30.0%
>2,500	15	9.4%
Size of town of practice		
<5,000	71	44.4%
5,000–20,000	36	22.5%
20,000–100,000	9	5.6%
>100,000	42	26.3%
Missing	2	1.2%

### 3.1 GPs familiarity with and current use of social prescribing

A total of 114 participating GPs (71.3%) reported being unfamiliar with SP. After receiving a brief explanation and an example scenario, 87 respondents (54.4%) indicated that they already incorporate some form of SP into their routine practice. When asked about specific SP approaches, more respondents reported using these practices than reported using SP overall. The most frequently reported approach was *referral to institutions or counseling services with patient-initiated contact* (*n* = 120; 75.0%), suggesting that slightly more GPs endorse the practice than knowing the concept. Other commonly reported approaches included the *distribution of brochures and informational materials* (*n* = 117; 73.1%), and *providing in-practice counseling during consultations* (*n* = 112; 70.0%). Direct *referral of patients to social prescribing activities* was reported by 88 respondents (55.0%).

Less commonly reported approaches included *referral to an existing external link worker* (*n* = 35; 21.8%), *nursing staff with an additional link worker function* (*n* = 23; 14.3%), and *referral to an existing link worker within the practice* (*n* = 13; 8.1%). The use of an *online platform listing social prescribing activities* was reported by 10 (6.3%) respondents.

Regression analysis indicated regional variation in SP use across Austria, with differences between federal states accounting for nearly 10% of the variance. The variance of the random intercept was 0.0928 (SD = 0.3046). Regarding state-level distributions, SP use was approximately balanced in Lower Austria (yes: 16, no: 13) and Vienna (yes: 9, no: 12), whereas in Styria more respondents reported using SP than not (yes: 31, no: 16). The sample was overrepresented in some federal states (e.g., Styria) and underrepresented in others (e.g., Vienna) compared with the overall population distribution. No systematic associations were observed between SP use and age, sex, or size of town (see Table 2).

**Table 2:** Regression analysis indicated regional variation in SP usage across Austria.

Variable	Coefficient	Standard error	*p*-value	Odds ratio (CI 95%)
Intercept	0.475	0.405	0.240	1.608 (0.728, 3.554)
Female	0.022	0.334	0.947	1.023 (0.531, 1.969)
35–45 years old^a^	−0.468	0.477	0.326	0.626 (0.246, 1.593)
46–55 years old^a^	0.126	0.383	0.742	1.134 (0.536, 2.401)
>5.000–20.000 inhabitants^b^	−0.418	0.421	0.320	0.659 (0.289, 1.501)
>20,000–100,000 inhabitants^b^	−0.644	0.433	0.137	0.455 (0.109, 1.9)
>100.000 inhabitants^b^	−0.787	0.729	0.280	0.525 (0.225, 1.227)

a Reference category: age group > 55 years.

b Reference category: municipalities with <5,000 inhabitants.

### 3.2 GPs perspectives on implementing SP

To assess general practitioners’ readiness for SP, we first examined their current satisfaction in addressing patients’ social concerns and their preferred referral options, followed by perceived barriers to implementation. Currently, only 60 GPs (37.5%) reported being satisfied with their ability to support patients with social concerns. In contrast, nearly all respondents expressed a willingness to adopt SP in daily practice (*n* = 154; 96.3%), particularly *referral to a contact person or link worker within their own practice* (*n* = 143; 89.4%), followed by *providing information/contacts to institutions for patient self-contact* (141; 88.1%), *nursing staff with an additional link worker function* (137; 85.6%), and *referral to an external link worker* (127; 79.4%). *Referral to telephone counseling services* was also commonly favored (132, 82.5%). In contrast, options relying on online tools or GP-initiated direct contact were less popular. Having an *online platform listing SP activities* was mentioned by 90 respondents (56.3%), a *hotline through which programs are referred* by 82 (51.3%), and *direct contact by the GP with institutions* by only 67 respondents (41.9%).

The most prominent perceived barriers to implementing SP related to financial resources, referral infrastructure, and workforce capacity. When combining “applies” and “rather applies,” a *lack of reimbursement for extended physician consultations* was most frequently reported (*n* = 138; 86.3%), followed by a *lack of referral structures* and insufficient *funding for additional staff* (each *n* = 131; 81.9%). A *lack of time in consultations* and concerns about *additional workload for already-employed staff* were also commonly reported (both *n* = 125; 78.1%). Knowledge-related barriers were less prominent, with a *lack of knowledge about existing social prescribing activities* reported by 117 respondents (73.1%). *Limited availability of, or lack of space for, link workers* was perceived as a barrier by 95 respondents (59.4%). In contrast, a *lack of understanding of social prescribing or link working* was reported less frequently (*n* = 51; 31.9%), while a *lack of consensus within the practice team* was the least commonly perceived barrier (*n* = 31; 19.4%).

### 3.3 Perceived impact of social prescribing

This section presents GPs’ perceptions of the relevance and impact of SP in daily practice, including its perceived effects on patient care and social issues commonly encountered in primary care. An overwhelming majority of respondents (*n* = 156; 97.5%) considered SP to be a meaningful tool in daily practice. This perception aligns with the high frequency with which GPs reported encountering patients’ social concerns. More than one-third of respondents (*n* = 61; 38.1%) reported addressing social concerns more than three times per week, and another third (*n* = 52; 32.5%) reported facing them two to three times per week. Among the 160 participating GPs, nearly half (43.8%) reported spending 10–20% of their working time on patients´ social concerns, about a quarter (21.9%) spent between 21 and 30, and 16.9% reported devoting more than 30% of their time to addressing patients´ social issues.

### 3.4 Perceived impact on patient care

GPs generally perceived SP as highly beneficial for patient care. Most strongly or moderately agreed that SP could *improve patients’ mental health* (95.7%, *n* = 153) and *reduce feelings of loneliness* (96.1%, *n* = 154). Moreover, they saw a potential positive impact of SP on *patient satisfaction* (95.6%, *n* = 153) and *overall patient care* (91.2%, *n* = 146), highlighting SP’s broad perceived value. Regarding perceived reductions associated with SP, GPs reported that SP could reduce their number of consultations (78.1%, *n* = 125) and, even more notably, consultations with other healthcare professionals (83.1%, *n* = 133). In addition, 74.4% (*n* = 119) expected SP to lead to fewer medication prescriptions, and 67.5% (*n* = 108) anticipated a reduction in their own workload.

### 3.5 Perceived impact on social issues encountered in practice

In total, *mental strain/feeling overwhelmed* was the most commonly reported social concern of patients encountered by GPs in daily practice (95.6%, *n* = 153) in daily practice. This was closely followed by *stress at work or stress due to unemployment* (94.4%, *n* = 151), and *caregiving for relatives* (90.0%, *n* = 144). Further social issues encountered frequently by GPs were *illness or death of relatives or friends* (80.0%, *n* = 128), *loneliness* (77.5%, *n* = 124), and disputes with a close person (75.6%, *n* = 121). The least frequently encountered concerns were *problems with housing or homelessness* (13.8%, *n* = 22) and *abuse or domestic violence* (6.3%, *n* = 10).

SP was perceived by GPs as potentially most beneficial for *loneliness* (96.9%, *n* = 155), *caregiving for relatives* (93.8%, *n* = 150), and *illness or death of relatives or friends* (92.5%, *n* = 148). High benefit was also perceived for *mental strain/feeling overwhelmed* (90.6%, *n* = 145), *disputes with a close person* (73.1%, *n* = 117), and *discrimination/exclusion* (73.1%, *n* = 117). Less frequently identified areas of potential benefit included *abuse or domestic violence* (67.5%, *n* = 107), *social legal issues* (63.1%, *n* = 101), *financial problems or poverty* (54.4%, *n* = 86), and *problems with housing or homelessness* (50.0%, *n* = 80).

**Figure 1:**
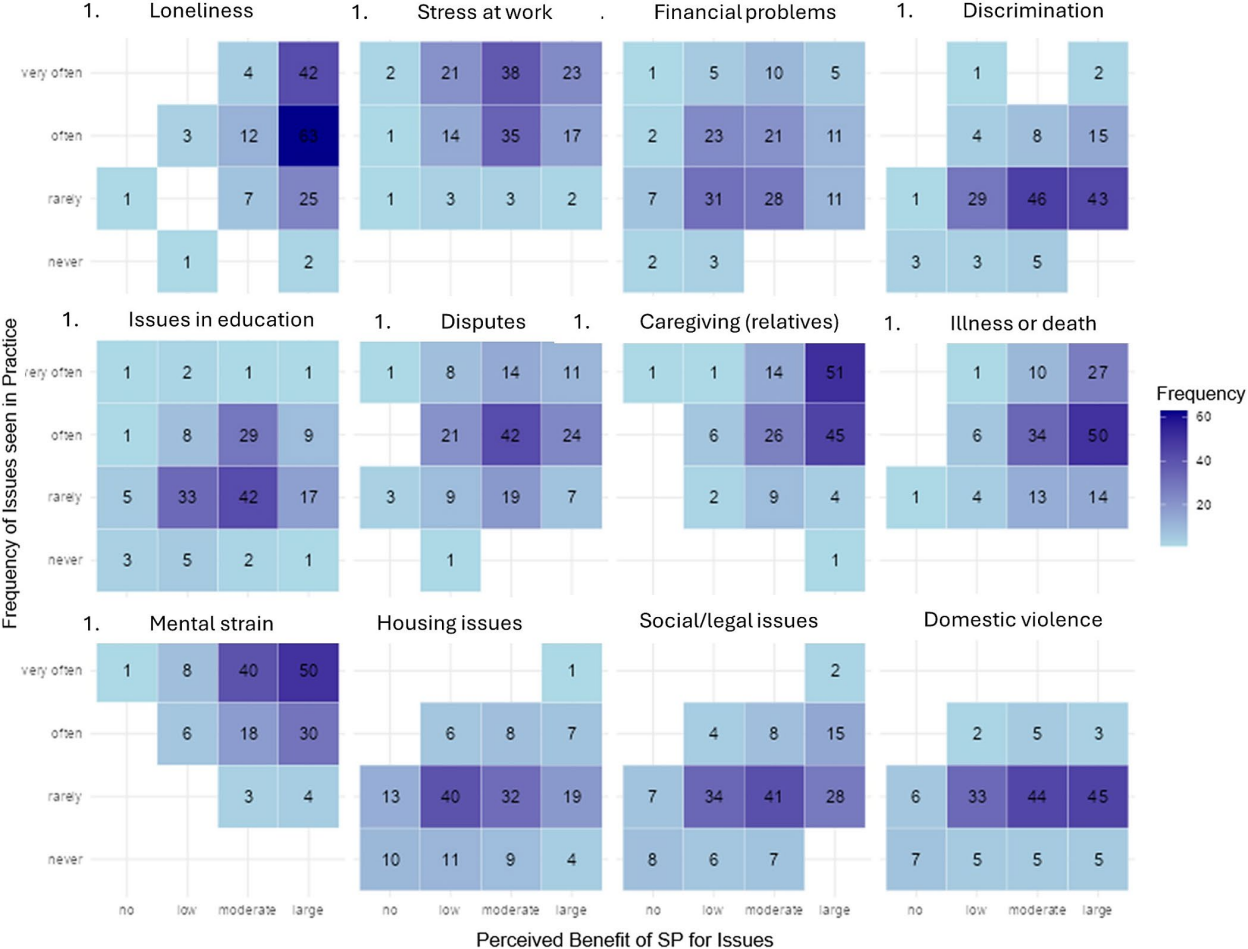
Associations between issue frequency and SP impact perception among GPs. Each panel shows one issue category. The y-axis indicates frequency (never to very often), the x-axis perceived impact (none to high). Cell values are response counts; darker colors indicate higher frequencies.

We analyzed associations visualized as a heat map to explore the perceived effectiveness of SP in addressing the social issues encountered by GPs in everyday practice (Figure 1). This provides insight into whether the social issues GPs believe are most responsive to intervention through SP are also those most frequently seen in practice. The heat map shows that some social issues encountered more frequently in practice were generally perceived by GPs as more likely to benefit from SP. Frequently encountered psychosocial issues—such as *loneliness*, *mental strain*, *caregiving for relatives*, and *illness or death of relatives or friends*—were consistently associated with moderate to large perceived benefit of SP. In contrast, less frequently encountered and more structural issues, including *financial problems*, *housing issues*, and *social or legal concerns*, were mainly linked to low to moderate perceived benefit. Overall, GPs perceived SP as most effective for common psychosocial concerns and less effective for infrequent or structural social problems. The visualization is shown in Figure 1.

## 4 Discussion

This cross-sectional study found that although most GPs were unfamiliar with the term SP prior to participation, many were already incorporating elements of SP into routine practice without formally identifying them as such. GPs were generally dissatisfied with their ability to address patients’ nonmedical needs but showed a strong willingness to adopt SP. Key barriers to implementation were primarily related to financial resources and formal referral pathways, followed by time and workload constraints. Overall, GPs considered SP a meaningful and potentially beneficial tool, particularly for addressing common psychosocial issues such as loneliness and mental health problems.

Using the same questionnaire as a study conducted in Germany (18), this study found that GPs in Austria appear to be somewhat more familiar with SP compared to German GPs. This may partly be explained by ongoing pilot projects in Austria since 2021 (13). Evidence from a European survey across 33 countries is limited for Austria, as only two Austrian GPs participated and the country was grouped among those with fewer than five responses (19). In contrast, in the same European study, approximately seven out of ten German GPs reported having heard the term SP. Overall, 56% of GPs across Europe were familiar with SP and 59% understood the concept, although awareness and implementation varied considerably between countries (19).

While many GPs were initially unfamiliar with the term *social prescribing*, more than half reported engaging in SP-like activities once the concept was explained. These activities, however, were predominantly delivered informally, with patients directed to services and expected to initiate contact themselves, or through in-practice counseling provided directly by GPs. In contrast, formal referral pathways involving link workers appear less common, and were likely tied to participation in funded pilot projects, suggesting that such pathways remain dependent on dedicated funding initiatives or broader national rollout efforts (19, 20).

The current model of SP in Austria places strong emphasis on formal link worker consultations, based on the argument that these are better suited to reaching populations with lower health literacy (17) and individuals who lack the confidence to access services on their own initiative (21). Additionally, signposting through leaflets is less likely to involve follow-up and/or feedback, resulting in more limited sustained engagement (2). However, signposting continues to represent a substantial, albeit largely hidden, form of SP activity—even in countries such as the UK, where SP has been implemented at a national level—reflecting the fact that clinicians have long engaged in signposting practices prior to the formalization of SP (22). This suggests that informal SP activities should not be dismissed given their continued widespread use. Rather, they can be understood as part of a broader continuum of practice, particularly suited to individuals who may benefit from brief information provision or in situations requiring greater flexibility in referral processes (23).

While our results did not find systematic associations between SP use and GP characteristics, findings from Germany interestingly showed that GPs in smaller towns were significantly more likely to adopt SP (18), potentially reflecting stronger personal connections between GPs and local organizations. It should be noted, that our sample size varied across the Austrian federal states, which may have influenced the robustness of our results.

Many GPs in this study reported dissatisfaction with their ability to support patients with nonmedical problems, particularly given the amount of consultation time such issues require. Although most GPs recognized the value of SP and expressed willingness to engage with it, they generally favored formalized referral pathways—most notably via link workers embedded within or connected to their practice. This is echoed in other studies, which show that while GPs view integrated models of care as meaningful, they tend to favor “outsourced” support structures for addressing patients’ social needs (18, 24, 25).

Our findings on barriers to the implementation of SP are broadly consistent with previous research. A systematic review (26) similarly identified key system-level constraints as key barriers, including unstable or short-term funding or poorly coordinated referral structures, and limited workforce capacity. Evidence from Germany (18) likewise highlighted practical implementation barriers such as limited consultation time, limited knowledge of SP, and insufficient funding for additional staff. In addition, Ajibade et al. (25) reported negative perceptions of SP and limited understanding of its practical implementation. Overall, our findings suggest that SP has the potential to be accepted within primary care settings, but remains insufficiently embedded into routine practice due to structural constraints—particularly inadequate financial support, weak referral infrastructure, and workforce capacity limitations.

GPs identified mental strain, work-related stress, caregiving responsibilities for relatives, and loneliness as the most common social concerns among patients. These issues have also been highlighted in previous studies (7, 18, 27). In contrast, financial problems, which were identified as the most prominent concern in some of these studies (7, 27), were less evident in our sample. Our findings further suggest that GPs perceive SP as highly beneficial for patient care and satisfaction, particularly for addressing the most commonly reported psychosocial concerns. This aligns with previous evidence indicating that SP can improve outcomes for patients experiencing these issues (28–30).

### 4.1 Strengths and limitations

The study provides valuable insights into GPs’ perspectives on SP in Austria. The results are particularly relevant for informing the further development and implementation of SP initiatives. A key methodological strength is the use of a survey instrument closely aligned with that used in Germany (18), facilitating meaningful cross-country comparisons. Moreover, several limitations should be noted. First, the low response rate (4.2%) may have introduced non-response and self-selection bias, and the reasons for non-participation cannot be determined due to the anonymous study design. Although the sample size was adequate for analysis, generalizability may be limited. Second, the use of self-reported data introduces potential bias, as GPs may overestimate their willingness to adopt SP, particularly if they have limited practical experience with formal SP pathways. Third, at the time, we did not ascertain which respondents had participated in official SP pilot projects, which may have influenced their perceptions and responses. Fourth, limited statistical power prevented inclusion of all relevant level-2 variables (e.g., size of practice or size of town) and a fully reliable estimation of the variance between the nine federal states and the circa 10% ICC in the regression analysis. Finally, variation in level-2 variables may be constrained due to an uneven distribution of cases among Austrian federal states.

## 5 Conclusion

This study found that although the majority of participating Austrian GPs were unfamiliar with the term SP, more than half reported incorporating SP elements such as signposting and direct referrals into their daily practice without formally labeling them as such. GPs were generally dissatisfied with their current capacity to adequately address patients’ social concerns. Accordingly, they expressed a strong willingness to adopt more formalized SP pathways, particularly when responsibilities for addressing social needs could be delegated to staff serving a link worker function.

However, significant barriers remain, including limited knowledge of existing SP activities and referral pathways, as well as insufficient structural support and service availability. Nevertheless, GPs perceived SP as a meaningful and promising approach, especially for addressing common psychosocial issues such as loneliness, work-related stress, and mental strain. GPs also anticipated that a structured implementation of SP could lead to improvements in patient care and satisfaction.

As national attention to SP continues to grow in Austria, including through emerging funding initiatives, future efforts should focus on increasing awareness and training for GPs, strengthening link worker infrastructure, and expanding the availability and visibility of community-based services. Addressing these gaps will be essential for the successful and sustainable integration of SP into routine primary care practice.

## Data Availability

The datasets presented in this article are not readily available because the datasets used and/or analyzed during the current study are available from the corresponding author on reasonable request. Requests to access the datasets should be directed to Christina Radl-Karimi, christina.radl-karimi@medunigraz.at.
