# Supplementary material for: General practitioners’ awareness, use, and perceptions of social prescribing in Austria
Source: Front Public Health. 2026 Jul 15;14:1897020. doi: 10.3389/fpubh.2026.1897020 (PMC13426155; doi:10.3389/fpubh.2026.1897020)
Supplement: Supplementary file 1 [file Data_Sheet_1.PDF]

Supplementary Material

Supplementary Table 1. Type of social prescribing performed in daily practice

Do you perform any of the following types of social prescribing in your practice?	Frequency
Direct referral to social prescribing activities in the patient consultations	88
Providing in-practice counselling during patient consultation	112
Providing contacts to institutions/counseling services with patient-initiated contact	120
Providing brochures/contacts/information	117
Referral of patients to an external *link worker*	35
Referral of patients to an existing *link worker* within their own practice	13
Referral to an online platform with listed *social prescribing* activities	10
Referral to nursing staff with additional *link working* function	23
Other	1

Supplementary Figure 1. Social prescribing across federal states in Austria

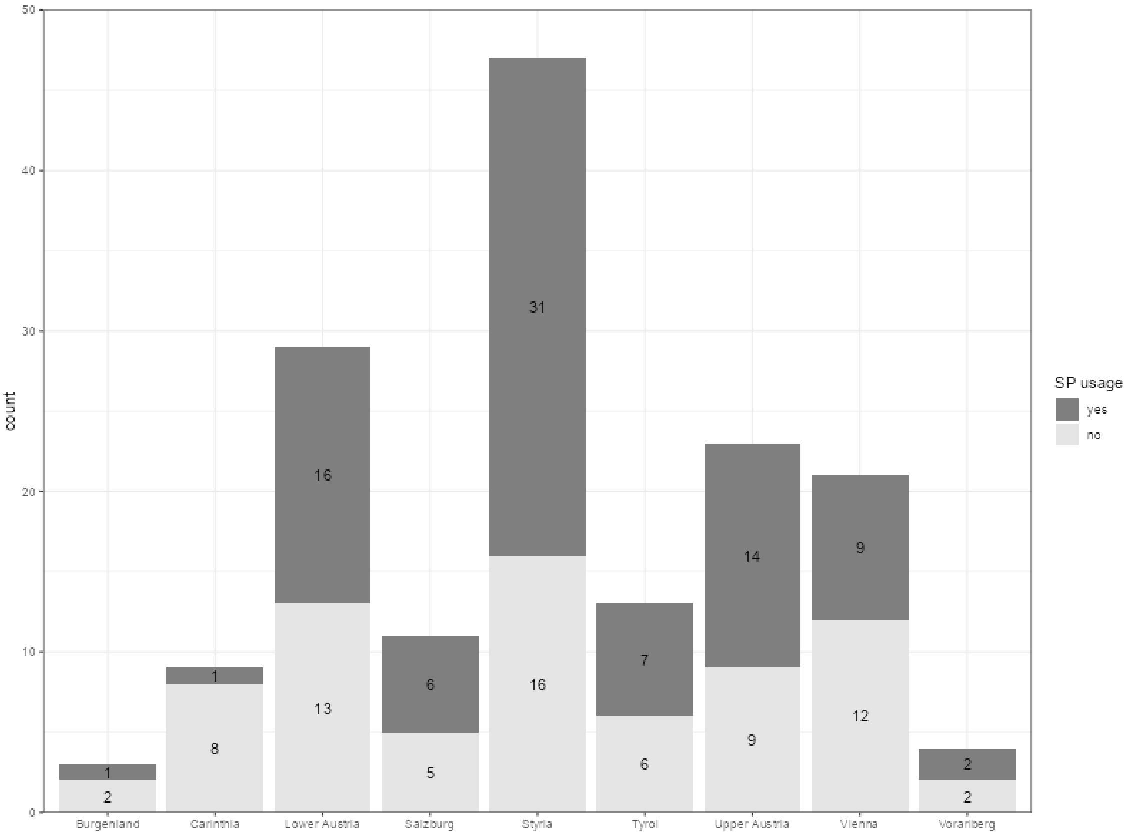

Supplementary Table 2. Social care structures preferred by GPs

**What type of referral to social care structures would you like to use as a general practitioner?**	**Does not apply**	**Rather does not apply**	**Rather applies**	**Applies**
Referral of patients to an external *link worker*	11	22	42	85
Contact person/*link worker* in their own practice (e.g., social worker)	7	10	47	96
Nursing staff with additional *link worker* function	8	15	54	83
Online platform with listed *social prescribing* activities/cooperation partners from the third sector	25	45	49	41
Hotline through which programs for patients are referred – you, as the general practitioner, establish contact	26	52	43	39
Providing information/contacts to various institutions for patient-initiated contact	4	15	61	80
Referral to telephone counselling services for patient-initiated contact	9	19	69	63
Direct contact by the general practitioner with institutions	32	61	39	28

Supplementary Table 3. Perceived barriers for implementing SP

**What do you currently see as the biggest barriers to implementing social prescribing?**	**Does not apply**	**Rather does not apply**	**Rather applies**	**Applies**
Lack of time in consultations	12	23	41	84
Funding for additional staff	11	18	25	106
Additional workload for already employed staff	9	26	44	81
Lack of knowledge about existing *social prescribing* activities	9	34	61	56
Lack of structures for referrals	9	20	44	87
Lack of reimbursement for extended physician consultations	11	11	34	104
Lack of consensus in the practice team (Mult-professionality)	76	53	21	10
Limited availability/lack of room for *link workers*	37	28	39	56
Lack of understanding of *social prescribing*/*link working*	62	47	28	23

Supplementary Table 4. Perceived frequency of patients´ social concerns in general practice

How many times a week are you confronted with patients' social concerns?	
**Times a week**	**Frequency**
Less than once a day	17
Once a day	30
2 – 3 times a day	52
More than 3 times a day	61

Supplementary Table 5. Perceived time spent on patients' social concerns in general practice

How much of your working time as a general practitioner is spent on the social concerns of your patients?	
**Part of working time**	**Frequency**
< 10%	28
10 – 20%	70
21 - 30%	35
> 30%	27

Supplementary Table 6. Perceived impact of social prescribing (improvement)

Social prescribing leads to an improvement in…				
**Outcomes**	**Does not apply**	**Rather does not apply**	**Rather applies**	**Applies**
Patient care	9	57	5	89
Patient satisfaction	3	50	4	103
Patients' feeling of loneliness	1	41	5	113
Patients' mental health	3	39	4	114

Supplementary Table 7. Perceived impact of social prescribing (reduction)

Social prescribing leads to a reduction in…				
**Outcomes**	**Does not apply**	**Rather does not apply**	**Rather applies**	**Applies**
Number of consultation reasons in your own practice	28	78	7	47
Number of consultation reasons in other healthcare facilities	20	82	7	51
Your own workload	37	62	15	46
Number of medication prescriptions	28	73	13	46

Supplementary Table 8. Perceived social issues commonly encountered in general practice

What social concerns/issues are most common in your practice?				
	**Never**	**Rarely**	**Often**	**Very often**
Loneliness	3	33	46	78
Stress at work/unemployment	0	9	84	67
Financial problems/poverty	5	77	21	57
Discrimination/exclusion	11	119	3	27
Issues in education/training	11	97	5	47
Dispute with a close person	1	38	34	87
Caregiving for relatives	1	15	67	77
Illness or death of relatives or friends	0	32	38	90
Mental strain/overwhelm	0	7	99	54
Problems with housing/homelessness	34	104	1	21
Social legal issues	21	110	2	27
Abuse/domestic violence	22	128	0	10

Supplementary Table 9. Perceived benefit of social prescribing by social concern

In your opinion, for which social concerns/issues do you think social prescribing can achieve the greatest benefit?				
	**Never**	**Rarely**	**Often**	**Very often**
Loneliness	1	4	23	132
Stress at work/unemployment	4	38	76	42
Financial problems/poverty	12	62	59	27
Discrimination/exclusion	4	37	59	60
Issues in education/training	10	48	74	28
Dispute with a close person	4	39	75	42
Caregiving for relatives	1	9	49	101
Illness or death of relatives or friends	1	11	57	91
Mental strain/overwhelm	1	14	61	84
Problems with housing/homelessness	23	57	49	31
Social legal issues	15	44	56	45
Abuse/domestic violence	13	40	54	53

Questionnaire (English translation)

Only selected items were included in the analyses presented in this manuscript. Items marked with an asterisk were collected for exploratory purposes only and were not analyzed in the present study.

1. Introductory filter question	Yes	No
**Are you working as a general practitioner?**		

If option 2 is selected → end of questionnaire

2. Familiarity with *social prescribing*	Yes	No
**Are you familiar with the concept of *social prescribing*?**		

Explanation
Social prescribing is a treatment concept in which non-medical (especially psychosocial health) concerns of patients are addressed by the treating general practitioners.

With the help of *social prescribing*, primary care providers can either refer their patients to local services themselves or via a so-called *link worker*. The term *link worker* comes from the United Kingdom and refers to a specialized, often external specialist who offers individual needs- and resource-oriented support. However, the function of link working can also be taken over by other specialists from the general practitioner team.

Example
As a general practitioner, you have been responsible for years for a patient who has recently come to you more often without medical concerns. In conversation, she tells you that her husband died a year ago and that she has since withdrawn from society. They refer the patient to a suitable *social prescribing* activity with their own knowledge of local services available. As a result, the patient works at the community center every Wednesday and you see the patient in your practice less often because her overall well-being has increased.

3. Implementation of *social prescribing*	Yes	No
**Are you already implementing social prescribing in your practice?**		

4. Do you perform any of the following types of *social prescribing* in your practice? (Multiple answers possible)	Yes	No
**Referral of patients to an external *link worker***		
**Contact person/link worker (e.g. social worker) in their own practice**		
**Nursing staff with additional *link worker* function**		
**Providing contacts to institutions/counselling centers for patient-initiated contact**		
**Online platform with listed *social prescribing* activities**		
**Providing brochures/contacts/information**		
**Direct referral to social prescribing activities in the patient consultation**		
**Own counselling in the practice during patient consultations**		
**Other, namely:**		

5. Below you will find a list of measures that correspond to social prescribing. Which of these do you prescribe yourself? *	Never	Rarely	Often	Very often
**Counselling services**				
Advisory service for informal/family caregivers				
Family counseling				
Addiction counselling				
Social counselling<br>Legal counselling				
**Cognitive activities**				
Learning cafés				
Language courses				
Play groups				
Literature circles / reading groups				
Libraries				
**Health-promoting activities**				
Walking groups				
Animal-assisted activities				
Gardening				
Senior dance classes				
Sports club activities				
Nutritional counselling				
Cooking classes				
**Creative activities**				
Creative crafting groups				
Paining classes				
Music-related activities				
Guided museum visits				
Local cultural center activities				
**Support services for specific target groups**				
Services for informal/family caregivers				
Self-help groups				
Community services				
Childcare groups				
**Networking opportunities for specific target groups**				
Single-parent groups				
Women´s groups				
Youth groups				
Senior groups				
**Social activities**				
Storytelling cafés / Conversation cafés				
Community center activities				
Neighborhood networks				
Clubs and associations of all kinds				
**Other**				
**Other**				

6. Meaningfulness of SP	Yes	No

Do you think social prescribing is meaningful?		

7. Please evaluate the following statements regarding *social prescribing* outcomes	Does not apply	Rather does not apply	Rather applies	Applies
Social prescribing leads to an improvement in…				
Patient care				
Patient satisfaction				
Patients' feeling of loneliness				
Patients' mental health				
Social prescribing leads to a reduction in…				
Number of consultation reasons in your own practice				
Number of consultation reasons in other healthcare facilities				
Your own workload				
Number of medication prescriptions				

	Less than once a day	Once a day	2–3 times a day	More than 3 times a day
8. How many times a week are you confronted with patients' social concerns?				

	Less than 10%	10–20%	20–30%	More than 30%
9. How much of your working time as a general practitioner is spent on the social concerns of your patients?				

10. What social concerns/issues are <u>most common</u> in your practice?	Never	Rarely	Often	Very often
Loneliness				
Stress at work/unemployment				
Financial problems/poverty				
Discrimination/exclusion				
Issues in education/training				
Dispute with a close person				
Caregiving for relatives				
Illness or death of relatives or friends				
Mental strain/overwhelm				
Problems with housing/homelessness				
Social legal issues				
Abuse/domestic violence				

11. In your opinion, for which social concerns/ issues do you think *social prescribing* can achieve the <u>greatest benefit</u>?	None	Low	Moderate	High
Loneliness				
Stress at work/unemployment				
Financial problems/poverty				
Discrimination/exclusion				
Issues in education/training				
Dispute with a close person				
Caregiving for relatives				
Illness or death of relatives or friends				
Mental strain/overwhelm				
Problems with housing/homelessness				
Social legal issues				
Abuse/domestic violence				

12. What type of referral to social care structures would you like to use as a general practitioner?	Does not apply	Rather does not apply	Rather applies	Applies
Referral of patients to an external *link worker*				
Contact person/*link worker* in the own practice (e.g., social worker)				
Nursing staff with additional *link worker* function				
Online platform with listed *social prescribing* activities/cooperation partners from the third sector				
Hotline through which programs for patients are referred – you, as the general practitioner, establish contact				
Providing information/contacts to various institutions – the patient establishes contact themselves				
Referral to telephone counselling services – the patient establishes contact themselves				
Direct contact by the general practitioner with institutions				

Satisfaction of supporting patients	Yes	No
13. Are you satisfied with your ability to support your patients on social issues?		

Implementation wish of *social prescribing*	Yes	No
14. Would you like to implement social prescribing in your daily practice?		

Training courses for *social prescribing**	Yes	No
15. Would you be interested in attending training courses on *social prescribing* yourself?		

Training courses for *social prescribing**	Yes	No
16. Would you consider training on social prescribing to be useful for your staff?		

17. What do you currently see as the biggest barriers to implementing social prescribing?	Does not apply	Rather does not apply	Rather applies	Applies
Lack of time in consultations				
Funding for additional staff				
Additional workload for already employed staff				
Lack of knowledge about existing *social prescribing* activities				
Lack of structures for referrals				
Lack of reimbursement for extended physician consultations				
Lack of consensus in the practice team (Mult-professionality)				
Limited availability/lack of room for *link workers*				
Lack of understanding of *social prescribing/link working*				

Sociodemographic data

	Male	Female	Other	Prefer not to say
18. What is your gender?				

	Under 35 yrs	35-45 yrs	46-55 yrs	Over 55 yrs
19. What is your age group?				

	Less than 5 years	5–15 years	16–30 years	More than 30 years
20. How long have you been working as a GP?				

	Solo practice	Group practice	Primary care unit / primary care center
21. What type of practice do work in?			

	Number of GPs
22. How many GPs are currently working in your practice? (excluding physicians in training / residents)	

	Number of GPs
23. How many other healthcare staff members are currently working in your practice? (e.g. medical assistants, qualified nursing staff)	

24. In which federal state is your practice located?	
Vienna	
Lower Austria	
Upper Austria	
Salzburg	
Styria	
Carinthia	
Burgenland	
Tyrol	
Vorarlberg	

	<5000 inhabitants	5000-20000 inhabitants	20000-100000 inhabitants	>100000 inhabitants
25. What is the size of the town/city where your practice is located?				
